# Effect of Ashwagandha Withanolides on Muscle Cell Differentiation

**DOI:** 10.3390/biom11101454

**Published:** 2021-10-04

**Authors:** Jia Wang, Huayue Zhang, Ashish Kaul, Kejuan Li, Didik Priyandoko, Sunil C. Kaul, Renu Wadhwa

**Affiliations:** 1 AIST-INDIA DAILAB, DBT-AIST International Center for Translational & Environmental Research (DAICENTER), National Institute of Advanced Industrial Science & Technology (AIST), Tsukuba 3058565, Japan; wang-jia0819@aist.go.jp (J.W.); zhang-huayue@aist.go.jp (H.Z.); ashish-kaul@aist.go.jp (A.K.); likejuan2005@sina.com (K.L.); didikpriyandoko@gmail.com (D.P.); s-kaul@aist.go.jp (S.C.K.); 2 College of Life Science, Sichuan Normal University, Chengdu 610066, China; 3 Department of Biology, Universitas Pendidikan Indonesia, Bangdung 40154, Indonesia

**Keywords:** Ashwagandha, withaferin-A, withanone, C2C12, muscle differentiation, protein aggregation, oxidative stress, autophagy

## Abstract

*Withania somnifera* (Ashwagandha) is used in Indian traditional medicine, Ayurveda, and is believed to have a variety of health-promoting effects. The molecular mechanisms and pathways underlying these effects have not yet been sufficiently explored. In this study, we investigated the effect of Ashwagandha extracts and their major withanolides (withaferin A and withanone) on muscle cell differentiation using C2C12 myoblasts. We found that withaferin A and withanone and Ashwagandha extracts possessing different ratios of these active ingredients have different effects on the differentiation of C2C12. Withanone and withanone-rich extracts caused stronger differentiation of myoblasts to myotubes, deaggregation of heat- and metal-stress-induced aggregated proteins, and activation of hypoxia and autophagy pathways. Of note, the Parkinson’s disease model of Drosophila that possess a neuromuscular disorder showed improvement in their flight and climbing activity, suggesting the potential of Ashwagandha withanolides for the management of muscle repair and activity.

## 1. Introduction

Ashwagandha (*Withania somnifera*, Solanaceae) is an Ayurvedic (Indian home medicine system) herb categorized as "rasayana" (possessing rejuvenating, longevity-enhancing, and revitalizing properties). It is commonly used for a spectrum of health-promoting effects including youthful vigor, activation of the immune and neuronal systems, muscle strength, and endurance. Trusted for its adaptogenic, cardiotropic, and cardioprotective effects, it is often marked as a health and brain tonic and used as a home-remedy for stress, frailty, anxiety, insomnia, nervous exhaustion, loss of memory, and cognitive disorders [1,2,3,4,5]. In spite of its extensive use, there are limited studies on the extraction of bioactive components from different parts of the plant that describe their mechanism(s) of action for the recognized/trusted bioactivities of Ashwagandha. Several recent studies have demonstrated that withaferin-A (Wi-A), withanolide-A (Wid-A), and withanone (Wi-N) are active ingredients in extracts prepared from the root, stem, and leaves of Ashwagandha. Wi-A was the first member of the withanolide (Wid) family to be isolated from the roots and is the most studied (in animal as well as cell culture experimental models) amongst several others including Wi-N, Wid-A, Wid-B, Wid-D, and their derivatives [6,7,8,9,10]. Wi-A has also been shown to possess a variety of health-promoting effects, including anti-inflammatory and anti-oxidative effects [3,11,12,13,14]. In mice models of ovalbumin (OVA)-induced airway inflammation, Wi-A caused inhibition of OVA-induced lung injury and fibrosis [15]. A study on the effects of Wi-A on experimentally induced cerebral infarction demonstrated a significant reduction in the infarct area and intimal hyperplasia. Molecular analysis revealed that it exerted neuroprotective effects by activating the PI3K/Akt pathway, modulating the expression of matrix metalloproteinases (MMPs), and inhibiting the migration of vascular smooth muscle cells (VSMCs) [16]. A large number of in vitro and in vivo studies have supported the anticancer activity of Wi-A and Wi-N and have also defined several molecular pathways for their action [17,18,19,20,21,22,23,24,25,26,27,28,29,30,31]. However, the cellular targets, the bioavailability, and the efficacy profiles for different cancer types and pharmacokinetics are yet to be resolved, in order to develop Wi-A as an anticancer drug. The anti-stress and anti-aging activities of Wi-N have been documented in cell-culture and mice experiments [32,33,34,35,36,37,38]. Studies on the animal models have also supported the anti-stress activity of Ashwagandha extracts. In a physical working capacity test of rats, Ashwagandha-extract-fed rats showed a significant increase in swimming endurance, relative heart weight, and glycogen content in the myocardium and the liver [39]. In a mouse model of Parkinson’s disease (PD), a neurodegenerative disorder that leads to impairment of balance and coordination, Wi-N-rich Ashwagandha-extract-treated mice showed an increase in the level of antioxidative enzymes and better performance of the treated group in all the physiological tests including grooming, rearing, narrow-beam walking, and foot slippery [40]. Thirunavukkarasu et al. [41] made an energy formula (EF) that contained Ashwagandha, caffeine and D-ribose and investigated its safety, cardioprotective ability, and energy impact in ischemic-reperfused myocardium model rats. They showed that EF-treated rats gained less body weight as compared to their corresponding control groups. Significant improvements in heart rate, coronary flow, aortic flow, left ventricular developed pressure and infarct size, levels of myocardial adenosine triphosphate, creatine phosphate, and phospho-adenosine monophosphate kinase levels were detected in rats subjected to global ischemia. Li et al. [42] showed the anti-obesity effect of Ashwagandha extract in a rat model. It was associated with improvement in the mitochondrial function of adipocytes and skeletal muscle. The study also showed that Wi-A promoted differentiation of pre-adipocytes into beige adipocytes and enhanced oxygen consumption in a C2C12 murine myoblast model. Azeemuddin et al. [43] investigated the effect of a herbal combination of *Boswellia serrata*, *Cissus quadrangularis*, and *Withania somnifera* on sarcopenia, which is the loss of skeletal muscle mass and strength as a result of aging. The evaluation of muscle mass, grip strength, motor coordination, gait, locomotor activity, and endurance in the control and test rat groups revealed a significant improvement in all the parameters. It was found that the herbal combination caused a reduction in the levels of TNF-alpha, IL-6, and myostatin while increasing the IGF-1 levels, suggesting that the active components in the combination have the potential to treat pathophysiological changes associated with sarcopenia. Maccioni et al. [44] recruited the amyotrophic lateral sclerosis (ALS) model of Drosophila to investigate the effect of *Mucuna pruriens* (Mp) and *Withania somnifera* (Ws). By electrophysiological and behavioral analyses, TDP-43 mutant flies were seen to have impaired climbing with unexpected hyperactivity and sleep dysregulation. Feeding the flies with Mp and Ws was shown to rescue these features, at least in part. Furthermore, flies exposed to the volatile anesthetics showed paradoxical responses that were partially normalized upon Mp or Ws treatment. De Rose et al. [45] characterized the effects of Mp and Ws on ALS-Drosophila and reported that Ws treatment significantly increased their lifespan and rescued climbing impairment. Similar studies using a Parkinson’s disease model of Drosophila also demonstrated the neuroprotective effects of Ws extract [46].

Several studies have reported the clinical efficacy of Ashwagandha extracts for management of body fat and muscles. A study on healthy volunteers reported a reduction in total- and LDL-cholesterol, an increase in muscle strength, and a reduction in fat [47]. Ziegenfuss et al. [48] reported that an aqueous extract of Ashwagandha improved upper- and lower-body strength, supported a favorable distribution of body mass, and was well-tolerated clinically in recreationally active men during the 12-week resistance training and supplementation period. A 16-week, randomized, double-blind, placebo-controlled, crossover study investigated the effects of Ashwagandha on fatigue, vigor, and steroid hormones in aging men (40–70 years old and overweight with mild fatigue) and reported increased levels of DHEA-S and testosterone [49]. The effect of Ashwagandha root extract consumption on muscle mass and strength in healthy young men engaged in resistance training was investigated in an eight-week, randomized, prospective, double-blind, placebo-controlled clinical study wherein muscle strength was kept as the primary efficacy and muscle size, body composition, serum testosterone levels, and muscle recovery were determined as the secondary efficacy measures. Interestingly, compared to the placebo subjects, the group treated with Ashwagandha had a significantly greater increase in muscle strength on the bench-press and the leg-extension exercises. Furthermore, a significant increase in muscle size at the arms and chest was observed in test groups that also showed a significant reduction in exercise-induced muscle damage and body fat percentage [50]. These studies have suggested the potential of Ashwagandha as a sports supplement/medicine and hence warrant molecular studies in muscle differentiation and stress pathways. Skeletal muscle differentiation is characterized by the expression of muscle-specific proteins, the withdrawal of cells from the cell cycle, and their fusion into multinucleated cells (myotubes) [51,52,53]. The characterization of proteins involved in muscle differentiation and their modulation by natural/synthetic drugs is valuable for understanding the biology of muscle disorders (including myopathies, muscular dystrophy, and spinal muscular atrophy) and their interventional therapies [51]. Here, we used C2C12 myoblasts (an easy and convenient system to study myocyte differentiation) and investigated their differentiation potential and stress tolerance in response to the treatment with Ashwagandha extracts, Wi-A, and Wi-N.

## 2. Materials and Methods

### 2.1. Preparation of Ashwagandha Withanolides

Withaferin-A (Wi-A) and withanone (Wi-N) were procured from Sigma-Aldrich, Japan. Dried leaf powder was used to prepare alcoholic (i-Extract), aqueous (M2-BCD, L7-BCD), and DMSO (M2-BDM, L7-DMSO, L7-BDM) extracts. Similar extracts were also prepared from dried stem powder by methods as described earlier [6,7,17,18].

### 2.2. Cell Culture

C2C12 (mouse myoblasts; ATCC-CRL-1772™) and U2OS (human osteosarcoma; ATCC-HTB-96™) cells were purchased from the National Institute of Physical and Chemical Research (RIKEN, Japan), and the Japanese Collection of Research Bioresources Cell Bank (JCRB, Japan), respectively. Cells were maintained in complete Dulbecco’s modified Eagle’s medium (DMEM; Life Technologies) supplemented with 10% fetal bovine serum (FBS) and 1% penicillin/streptomycin at 37 °C, 5% CO_2_, and 95% air in a humidified incubator. For induction of myogenic differentiation, C2C12 cells were cultured in DMEM with 2% horse serum (HS) and 1% penicillin/streptomycin (differentiation medium) after reaching ~75% confluency. Alternatively, cells were cultured at high density in DMEM-10% FBS. The extent of differentiation was observed under the microscope and scored for multinucleated (~2–8 nuclei) myotubes. Cells that showed poor differentiation were isolated by cloning cylinders. Cells were expanded and subjected to differentiation again in DMEM-2% HS and DMEM-10% FBS-high-density conditions in parallel. The effect of withanolides on cell viability was determined by MTT assays in which various concentrations of purified compounds and extracts were used, and the IC50 and IC10 (the concentration at which 50% and 10% of the cell population remained viable, respectively) were determined from the cell viability plots (Supplementary Table S1). The IC10 concentration was considered nontoxic and was used for the induction of differentiation.

### 2.3. HPLC Analysis

The contents of Wi-A and Wi-N in Ashwagandha withanolides were analyzed by reversed-phase HPLC using the Develosil C30-UG Column (Batch No. 030718; Nomura Chemical Co., LTD, Seto, Aichi, Japan). Reversed phase HPLC was carried out at a flow rate of 1 mL/min at a column temperature of 40 °C. Gradient extraction was carried out with water (Solution A) and ethanol (Solution B). The 30-min gradient program (Solution B-50% for 0.01 min, 50–80% for 25 min, and 50% for 5 min before stopping the pump) was applied. The detection of components in eluted fractions was carried out at 237 nm.

### 2.4. Muscle Cell Differentiation

C2C12 cells were plated at a density of 2 × 10^5^ per well in 6-well plates and was allowed to adhere to the substratum overnight. At ~75% confluency, cells were subjected to differentiation medium containing Ashwagandha withanolides (nontoxic concentrations 0.01 and 0.05 μg/mL for i-Extract, 0.001 and 0.005 μg/mL for Wi-A, and 0.01 and 0.05 μg/mL for Wi-N, as determined by independent cell viability assays) and were followed for the formation of myotubes for the next 2–11 days. The cell morphology and appearance of myotubes in the control and the treated cells was observed under a phase contrast microscope (Nikon Eclipse TE300; Nikon, Tokyo, Japan).

### 2.5. Western Blotting

The control and treated C2C12 cells were harvested by trypsinization and lysed in radioimmunoprecipitation assay buffer (RIPA buffer; Thermo Fisher Scientific, Waltham, MA, USA) supplemented with a protease inhibitor cocktail (Roche Applied Science, Mannheim, Germany). The concentrations of proteins in lysates were determined using the Pierce BCA Protein Assay kit (Thermo Fisher Scientific, Waltham, MA, USA). Lysates were subjected to SDS-polyacrylamide gel electrophoresis followed by transfer to a polyvinylidene difluoride (PVDF) membrane (Millipore, Billerica, MA, USA) using a semidry transfer blotter (ATTO Corporation, Tokyo, Japan). Membranes were blocked with 3% bovine serum albumin (BSA; WAKO, Osaka, Japan) at room temperature for 1 h followed by incubation with target protein-specific primary antibodies including anti-myogenin (556358; BD Biosciences, San Jose, CA, USA), anti-HIF-1 alpha (NB100-479; Novus biologicals, Littleton, CO, USA), anti-LC3B (2775s; Cell Signaling Technologies, Danvers, MA, USA), anti-Beclin1 (G-11; Santa Cruz Biotechnology, Paso Robles, CA, USA), anti-ATG5 (12994s; Cell Signaling Technologies, Danvers, MA, USA), anti-ATG16L1 (8089s; Cell Signaling Technologies, Danvers, MA, USA), and anti-p62 (ab56416; Abcam, Cambridge, MA, USA) at 4 °C overnight. The blots were incubated with the following secondary antibodies conjugated to horseradish peroxidase: anti-rabbit IgG (31460, Thermo Fisher Scientific, Waltham, MA, USA) or anti-mouse IgG (31430, Thermo Fisher Scientific, Waltham, MA, USA) and detected by enhanced chemiluminescence reaction (ECL) (GE Healthcare, Amersham, Buckinghamshire, UK). β-actin antibody (643807, BioLegend, Tokyo, Japan) was used to detect β-actin as an internal loading control. Quantitation of the protein expression was determined using ImageJ software (National Institute of Health, Bethesda, MD, USA).

### 2.6. Immunostaining

C2C12 cells (1 × 10^5^/well) were seeded on 18-mm glass coverslips placed in 12-well plates and allowed to adhere overnight. Subsequently, the culture medium was replaced with a differentiation medium containing Ashwagandha withanolides. Cells were fixed in methanol:acetone (1/1, *v*/*v*) at 4 °C for 10 min, permeabilized with Tween-20 in phosphate-buffered saline (PBST), blocked with 2% bovine serum albumin (BSA)/PBST for 1 h, and incubated with anti-myogenin primary antibody (556358; BD Biosciences, San Jose, CA, USA), anti-HIF-1 alpha (NB100-479; Novus biologicals, Littleton, CO, USA), and anti-LC3B (2775s; Cell Signaling Technologies, Danvers, MA, USA) at 4 °C overnight. The cells were then incubated with either Alexa-Fluor-488-conjugated goat anti-mouse IgG secondary antibody (A11029, Invitrogen Molecular Probes, Eugene, OR, USA) or Alexa-Flour-594-conjugated goat anti-rabbit IgG secondary antibody (A11012, Invitrogen Molecular Probes, Eugene, OR, USA) for 1 h and Hoechst 33342 (Sigma, St. Louis, MO, USA) in the dark for 10 min. The coverslips were inverted and mounted on glass slides and visualized under a Carl Zeiss microscope (Axiovert 200M; Tokyo, Japan). Myotubes with two or more nuclei were defined as cells positive for myogenin.

### 2.7. Protein Aggregation and De-Aggregation Assay

C2C12 cells (2 × 10^5^ cells/well) were plated in 6-well plates and allowed to adhere overnight. Cells were transfected with the plasmid expressing GFP driven from the β-actin promoter using Lipofectamine 2000 transfection reagent (11668027, Thermo Fisher Scientific, Waltham, MA, USA) in an Opti-MEM reduced serum medium (Gibco, 10149832, Thermo Fisher Scientific, Waltham, MA, USA). The transfected cells were stressed with sodium (meta)arsenite (NaAsO_2_, 20 μM) for 24 h, recovered in either the control or Ashwagandha withanolides-supplemented medium for 48 h, and then visualized under a fluorescent microscope. Aggregates were quantified using ImageJ software (National Institute of Health, Bethesda, MD, USA).

### 2.8. Heat-Induced Luciferase Folding Assay

C2C12 cells (2 × 10^5^ cells/well) were plated in 6-well plates and allowed to adhere to the substratum overnight. Cells were transfected with pGL4-p53-3′ UTR expressing luciferase driven by a constitutive promoter using Lipofectamine™ 2000 transfection reagent (11668027, Thermo Fisher Scientific, Waltham, MA, USA) in an Opti-MEM reduced serum medium (Gibco, 10149832, Thermo Fisher Scientific, Waltham, MA, USA). The transfected cells were heat-shocked at 42 °C and 5% CO_2_ for 2 h, followed by recovery at 37 °C either in the control or Ashwagandha withanolides-supplemented medium for 48 h. The transfected cells were lysed in passive lysis buffer for luciferase expression estimation using the Dual-Luciferase Reporter Assay System (Promega, Madison, WI, USA) following the manufacturer’s protocol. The luciferase activity was measured using a Tecan Infinite M200 Pro microplate reader (Tecan Group Ltd., Mannedorf, Switzerland).

### 2.9. HRE-Responsive Luciferase Reporter Assay

U2OS cells (2 × 10^5^ cells/well) were plated in 6-well plates and allowed to adhere to the substratum overnight. Cells were transfected with the plasmid expressing luciferase driven by a promoter containing hypoxia-responsive element (HRE) using Lipofectamine 2000 transfection reagent (11668027, Thermo Fisher Scientific, Waltham, MA, USA) in an Opti-MEM reduced serum medium (Gibco, 10149832, Thermo Fisher Scientific, Waltham, MA, USA). The transfected cells were treated with Ashwagandha withanolides for 48 h and lysed in passive lysis buffer for luciferase expression estimation using the Dual-Luciferase Reporter Assay System (Promega, Madison, WI, USA) following the manufacturer’s instructions. The luciferase activity was measured using a Tecan Infinite M200 Pro microplate reader (Tecan Group Ltd., Mannedorf, Switzerland).

### 2.10. Drosophila Climbing Activity Assay

The climbing assay (negative geotaxis assay) was performed to determine the locomotor ability of the control and the Ashwagandha-extract-fed Parkinson’s model of Drosophila [54]. The latter possessed locomotion malfunction like patients of this neurodegenerative disorder. The climbing activity assay was performed on three age groups (2-, 3-, and 4-weeks-old) treated with the control and the Ashwagandha leaf water extract (WEX) [7] (1% and 10%, respectively). Cohorts of 30 flies from each experimental group were subjected to the assay. The tested flies were placed individually in a vertically positioned plastic tube (length = 10 cm; diameter = 1.5 cm) and taped to the bottom. The climbing time (s) was recorded upon crossing a line drawn at 6 cm from the bottom. The number of flies that could climb up to, or above, this line within 10 s was recorded and expressed as a percentage of the total flies.

### 2.11. Statistical Analysis

All the experiments were performed in triplicates, and data were expressed as mean ± standard deviation (SD). Statistical analysis was calculated by an unpaired *t*-test (GraphPad Prism GraphPad Software, San Diego, CA, USA) and shown as * *p* < 0.05, ** *p* < 0.01, and *** *p* < 0.001.

## 3. Results

### 3.1. Isolation of C2C12 Clones with Weak and Uniform Differentiation Characteristics

C2C12 mouse myoblasts, when subjected to differentiation by (i) culture in a medium supplemented with 2% HS or (ii) high-density culture in a medium supplemented with 10% FBS showed heterogenous differentiation. In both cultures, 30–40% differentiation, multinucleated myotubes containing 2–8 nuclei, and undifferentiated cells were observed. We anticipated that such a heterogenous response will not be appropriate for investigating the effect of bioactive compounds of ashwagandha on the differentiation potential of myoblasts, and hence we first performed cloning by serial dilution. Forty-eight single-cell clones were subjected to three cycles of differentiation using 10% FBS high-density culture in 48-well cell-culture dishes. The clones that showed poor differentiation were carried forward and tested for their differentiation in 2% HS-supplemented medium. We isolated three clones that did not show differentiation into myotubes in FBS-supplemented medium; however, they showed slow and somewhat uniform differentiation in a medium supplemented with 2% HS. One of the three clones (C2C12-C3, called C3 hereafter) was selected for the present study (Supplementary Figure S1).

### 3.2. Effect of Ashwagandha Leaf Extracts and Purified Withanolides on C2C12 Differentiation

**Figure 1:**
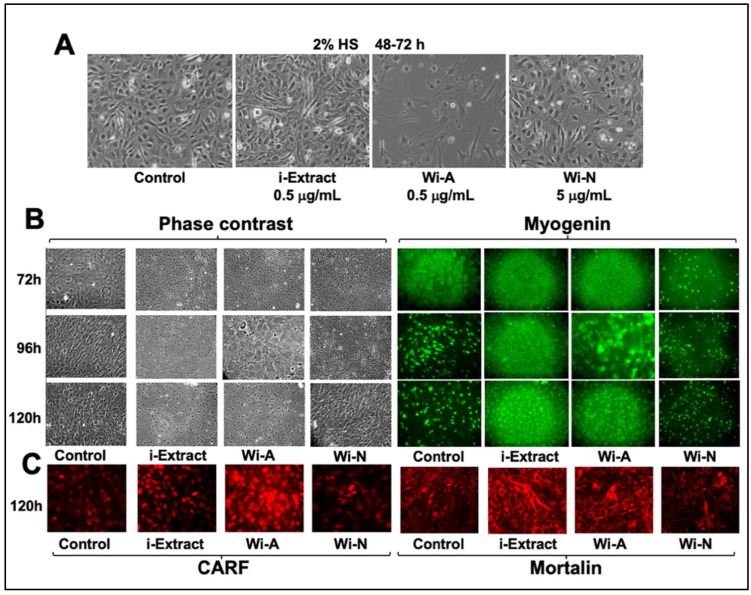
Effect of Ashwagandha leaf extract and purified withanolides on C2C12 differentiation. (**A**) C3 clone was differentiated in the control (2% HS) and the medium supplemented with bioactive compounds derived from Ashwagandha (i-Extract, Wi-A, and Wi-N). Phase contrast, myogenin (**B**), CARF, and mortalin immunostaining (**C**) of C3 clone treated with i-Extract, Wi-A, and Wi-N.

We next subjected the C3 clone to differentiation in either the control (2% HS medium) or the test medium supplemented with bioactive compounds derived from Ashwagandha (i-Extract [6]/Wi-N/Wi-A) at their nontoxic concentrations as determined by cell viability assays. As shown in Figure 1A, C3 clones showed negligible differentiation during 48–72 h culturation. On the other hand, i-Extract (0.5 µg/mL) and Wi-N (5 µg/mL) were well tolerated and exhibited some alignment of cells to multinucleated myotubes. Wi-A (0.5 µg/mL) showed cytotoxicity. Upon extended incubation for 5–6 days, we observed myotubes in the control and Wi-N-supplemented cultures (Figure 1B). The control and test cultures were subjected to myogenin (master regulator and biomarker for muscle cell differentiation) immunostaining. We found remarkable upregulation of myogenin expression by 96 h of treatment in the control culture. Whereas i-Extract and Wi-A were marked by cytotoxicity and poor expression of myogenin, the Wi-N-supplemented culture showed clear upregulation that initiated at 72 h post-treatment. Based on our earlier findings on the cytotoxicity of Wi-A and the anti-stress effect of Wi-N on human normal and cancer cells [6,17,18,32,33,36,55], we anticipated similar activities for C2C12 myoblasts. Indeed, the immunostaining of stress protein (CARF) in control and treated cells revealed its increase, signifying growth arrest in Wi-A-treated cells (Figure 1C) [56,57]. Similarly, immunostaining with anti-mortalin antibody showed a shift in the mortalin staining pattern from perinuclear (control cells) to pancytoplasmic in i-Extract- and Wi-A-treated cells, signifying senescence-like growth arrest in these cells, as reported earlier (Figure 1C) [28,58,59,60,61,62,63,64]. We next used a non-toxic concentration of i-Extract and Wi-A to perform time lapse observations on differentiation.

**Figure 2:**
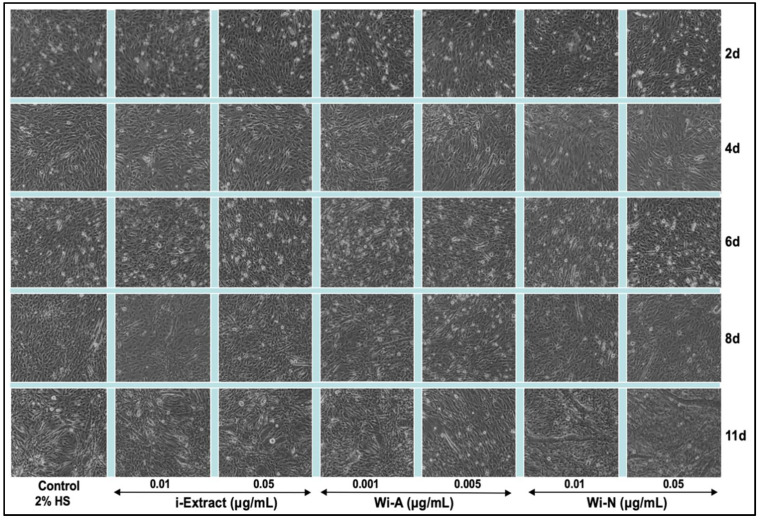
Time lapse observations on differentiation of C3 clone of C2C12 myoblasts treated with nontoxic doses of i-Extract, Wi-A, and Wi-N. i-Extract and Wi-A triggered some weak differentiation in C2C12 myoblasts; Wi-N-treated cells showed strong differentiation to myotubes.

As shown in Figure 2, the C3 clone showed poor differentiation in the eight-day-old control culture. On the other hand, the i-Extract and Wi-A cultures showed a ~30% differentiation and remained unchanged for the next two days examined. The Wi-N-treated culture showed about 50% differentiation by day 8 and remarkably increased to 70–80% by day 11. The results were confirmed with two concentrations (i-Extract: 0.01 and 0.05 μg/mL, Wi-A: 0.001 and 0.005 μg/mL, and Wi-N: 0.01 and 0.05 μg/mL) of each of the compounds. Based on these data, it was concluded that whereas i-Extract and Wi-A caused mild growth arrest in C2C12 cells, their low concentrations induced the withdrawal of cells from proliferation and triggered differentiation. Wi-N, on the other hand, was relatively safe and caused strong differentiation to myotubes.

**Figure 3:**
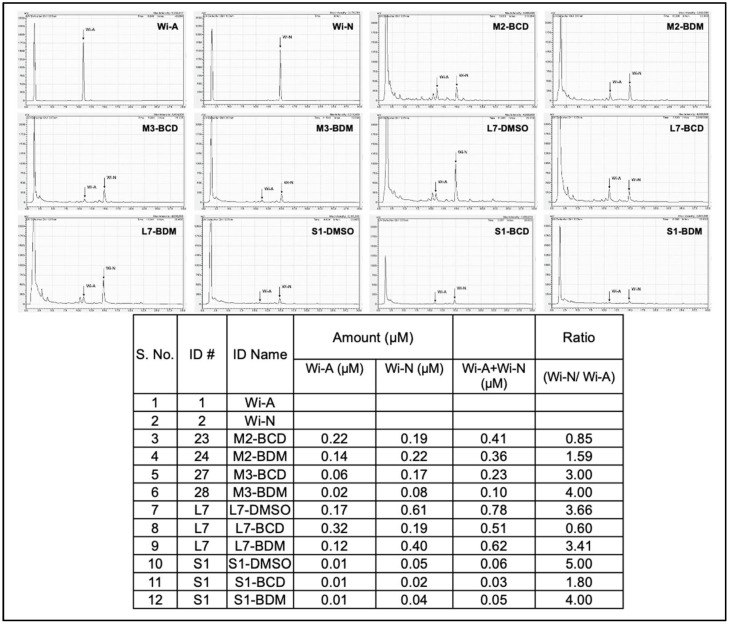
HPLC analyses for the contents of Wi-A and Wi-N in Ashwagandha leaf and stem extracts.

**Figure 4:**
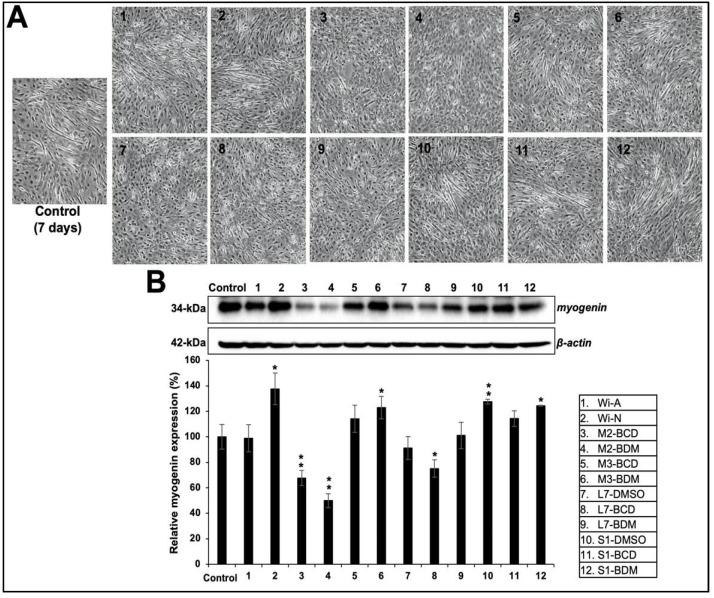
Effect of Ashwagandha extracts and purified withanolides on differentiation in C2C12 cells. (**A**) Phase contrast microscopic images showing the cell morphology and appearance of myotubes in control and treated cells. (**B**) Western blotting analysis for myogenin protein (master regulator and biomarker for muscle cell differentiation) after incubation of Ashwagandha withanolides. Quantitation of the results is shown below (mean ± SD, *n* = 3), * *p *< 0.05, ** *p *< 0.01 (Student’s *t*-test to control).

We had earlier established the methods to prepare water-based extraction of bioactive components from Ashwagandha leaves using cyclodextrin and were able to generate extracts either rich in Wi-A or Wi-N [7]. The content of Wi-A and Wi-N has also been shown to vary in different parts of the Ashwagandha plant; Wi-N seemed to be present in a high ratio in stems than in leaves [65]. In light of this information, we generated extracts from Ashwagandha leaves and stems using cyclodextrin. The insoluble fractions were dissolved in DMSO. The extracts were analyzed for the content of Wi-A and Wi-N by HPLC (Figure 3) and their effect on differentiation in the C3 clone cultured in a 2% HS-supplemented medium. The cells were treated with nontoxic doses (determined by independent dose-dependent cytotoxicity assays, Supplementary Table S1). We found that the extracts with a low content of major withanolides (Wi-A+Wi-N; 0.05 to 0.1 μM) and a high ratio of Wi-N:Wi-A (3 to 5) resulted in strong differentiation of the C3 clone as determined by the formation of myotubes observed under the microscope (Figure 4A). We also subjected the control and the treated cells to Western blotting analysis to examine the myogenin. As shown in Figure 4B, samples #2, #6, #10, and #12 caused higher induction of myogenin expression than the rest, in agreement with the stronger differentiation (observed under the microscope) in cells treated with these samples. These data indicated that the extracts with a high Wi-N:Wi-A ratio resulted in a stronger differentiation phenotype. On the other hand, extracts with a high Wi-A showed poor differentiation when used at the IC10 or even the IC1 concentration. Taken together, these data demonstrated that the treatment of C2C12 cells with the extracts containing a lower amount of total withanolides and a high ratio of Wi-N, in particular, promoted their differentiation to myotubes.

**Figure 5:**
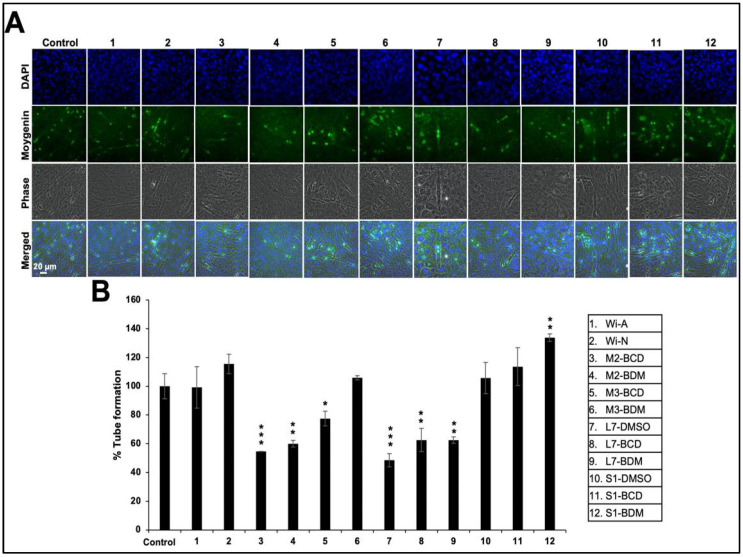
Induction of differentiation in response to Ashwagandha extracts and purified withanolides. (**A**) Immunostaining for myogenin protein after incubation of Ashwagandha withanolides. (**B**) Quantitation of the results is shown below (mean ± SD, *n* = 3), * *p* < 0.05, ** *p* < 0.01, *** *p* < 0.001 (Student’s *t*-test to control).

We next performed imaging analyses of differentiation and myogenin expression by immunostaining. As shown in Figure 5, it was revealed that the stem extracts that possessed a relatively high Wi-N:Wi-A ratio that caused strong induction of myogenin expression and differentiation.

### 3.3. Effect of Ashwagandha Extracts and Purified Withanolides on Metal and Heat-Shock-Induced Protein Aggregation

**Figure 6:**
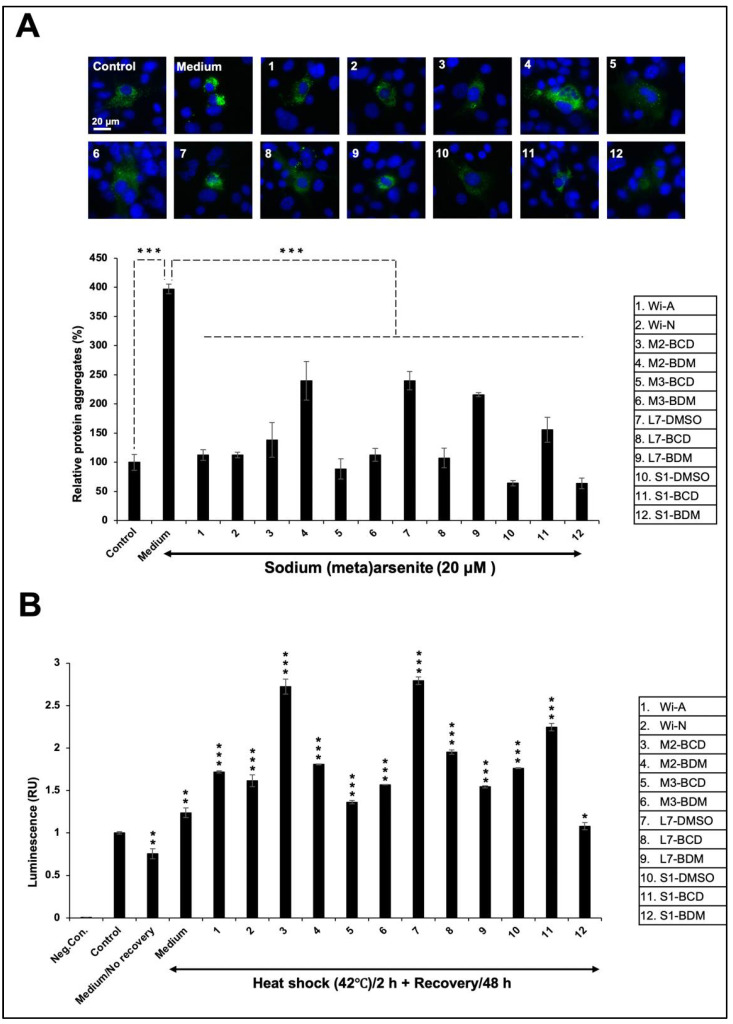
Effect of Ashwagandha extracts and purified withanolides on metal and heat-shock-induced protein aggregation. (**A**) Protein aggregation and deaggregation assay showing the GFP aggregation in sodium-(meta)arsenite-treated cells and deaggregation after incubation with Ashwagandha withanolides. (**B**) Luciferase activity in heat-shock was treated and recovered either in control or Ashwagandha-withanolides-supplemented medium. Quantitation of the results is shown below (mean ± SD, *n* = 3), * *p *< 0.05, ** *p* < 0.01, *** *p* < 0.001 (Student’s *t*-test).

Protein aggregation and the accumulation of molecular garbage is one of the causes of age-related decline in differentiation capacity. Skeletal muscle is one of the tissues that exhibits early age-related changes such as dysfunction and the loss of muscle mass. Studies in Drosophila have shown the progressive accumulation of protein aggregates in muscle that was associated with impaired muscle function. Furthermore, the proliferation and differentiation abilities of satellite cells of mature myofibers showed a decline with increasing age [66,67,68,69]. Therefore, we examined if Ashwagandha extracts could recover or reverse some of such damages by using two-model assay systems: (i) metal (NaAsO_2_)-induced aggregation of GFP protein and (ii) heat-induced folding of luciferase protein. Cells transfected with GFP and luciferase reporters were subjected to stress and subsequent recovery either in the control or Ashwagandha extracts/bioactive compounds-supplemented medium. As shown in Figure 6A, NaAsO_2_ caused the aggregation of GFP. Cells treated with the extracts and purified withanolides showed significant deaggregation of GFP. Of note, Wi-N and the extracts that contained a high amount of Wi-N as compared to Wi-A caused maximum deaggregation. Intriguingly, these effects matched with the differentiation potential of extracts. The quantitative measure of luciferase activity in cells subjected to heat shock revealed heat-induced misfolding/aggregation of luciferase protein. As shown in Figure 6B, heat shock caused a ~20–30% decrease in luciferase activity in the control cells. On the other hand, the treated cells showed an increase in luciferase activity. In contrast to the GFP aggregation assay, luciferase activity was increased in extracts #3, #7, and #11 that possessed relatively high levels of Wi-A. These data demonstrated that the bioactive compounds of Ashwagandha extracts protected the cells against stress-induced aggregation of proteins.

### 3.4. Effect of Ashwagandha Extracts and Purified Withanolides on Hypoxia and Autophagy

Oxidative stress in skeletal muscle has been shown to regulate muscle differentiation and functional characteristics. With low to moderate levels of oxidative stress, p53 is involved in activating pathways that prolong the time for cells to repair by activating cell cycle arrest and autophagy and enhancing cell survival. However, with higher levels of stress intensity and duration (including irradiation, hypoxia, and oxidizing agents) it causes apoptosis, and hence, p53 acts as a threshold regulator of cellular homeostasis [70]. Hypoxia-inducible transcription factor (HIF-1α) is the master regulator of hypoxia signaling. Deregulated HIF-1α signaling has been associated with several pathological conditions including cancers and brain- and muscle-disorders. Whereas under normoxia conditions, HIF-1α undergoes hydroxylation and degradation by the proteasome-mediated degradation pathway, hypoxia prevents HIF-1α hydroxylation and degradation [71]. As a result, HIF-1α accumulates, translocates into the nucleus, dimerizes with HIF-1β, and transactivates several effector proteins involved in cancer cell migration and angiogenesis.

**Figure 7:**
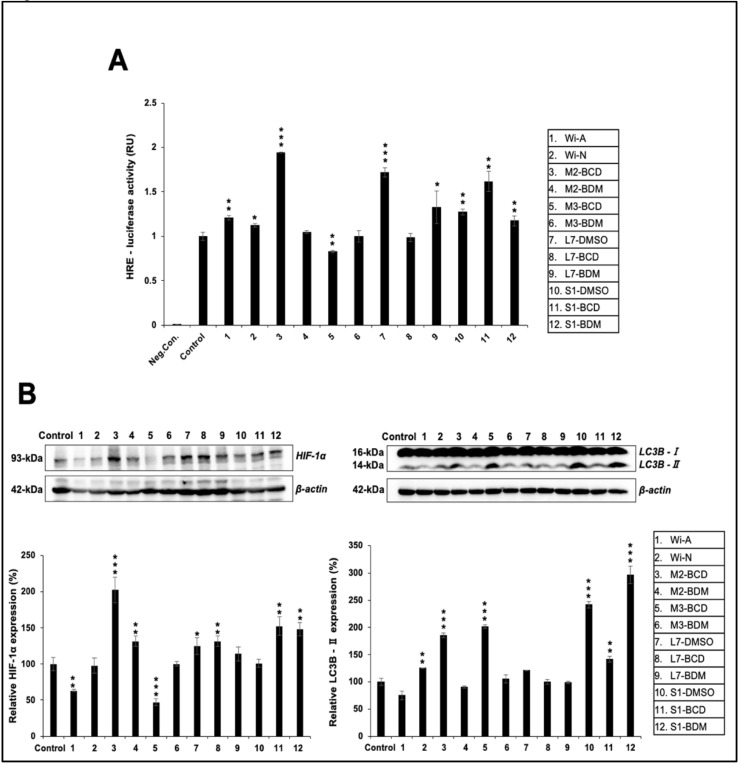
Effect of Ashwagandha extracts and purified withanolides on hypoxia and autophagy. (**A**) HRE-promoter-driven luciferase assay in control and treated cells. (**B**) Western blotting analysis for HIF-1α and LC3B proteins after treatment with Ashwagandha withanolides. Quantitation of the results is shown below (mean ± SD, *n* = 3), * *p *< 0.05, ** *p* < 0.01, *** *p* < 0.001 (Student’s *t*-test to control).

**Figure 8:**
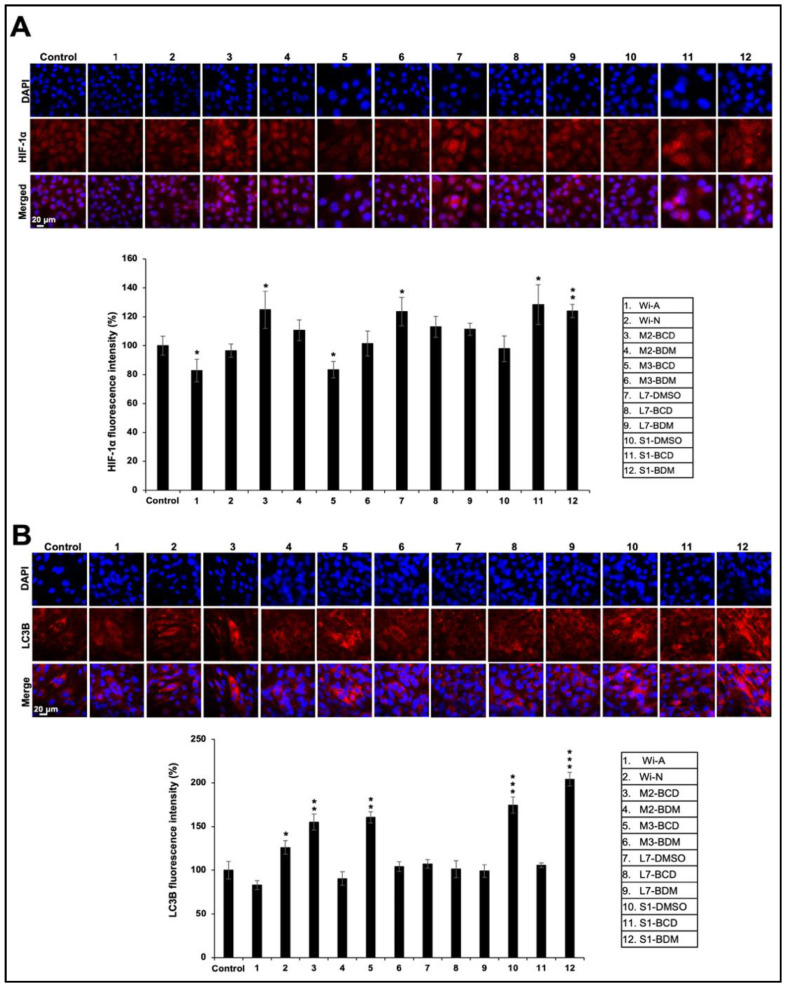
Immunostaining of HIF-1α (**A**) and LC3B (**B**) proteins in Ashwagandha withanolides-treated C2C12 cells. Quantitation of the results is shown below (mean ± SD, *n* = 3), * *p* < 0.05, ** *p* < 0.01, *** *p* < 0.001 (Student’s *t*-test to control).

We investigated the effect of Ashwagandha extracts and the purified withanolides on hypoxia responsive element (HRE)-luciferase activity. Cells transfected with plasmid expressing HRE-driven luciferase were subjected to control and Ashwagandha extracts/bioactive compounds-supplemented medium. As shown in Figure 7A, HRE promoter-driven luciferase assay showed a stronger increase in cells treated with extracts #3, #7, and #11, which contained a relatively high content of Wi-A as compared to other extracts and Wi-N. This result was in line with the data obtained from the recovery of heat-induced folding of luciferase. Detection of HIF-1α protein by Western blotting using anti-HIF-1α antibody also exhibited an increase in cells treated with #3, #7, and #11 (Figure 7B). In addition, samples #4, #8, and #12 that were marked by a high Wi-N content also showed an increase in HIF-1α. Consistently, the expression of HIF-1α protein, as detected by immunostaining, showed an increase in the treated samples: #3, #4, #7, #8, #9 #11, and #12 (Figure 8A).

Differentiation of myoblasts requires functional degradative systems including autophagy that assist in the formation of multinucleated terminally differentiated myotubes. The upregulation of proteins (LC3B-II, BECN1 (Beclin 1), ATG7, and ATG12-5) involved in autophagy has been reported during C2C12 differentiation. Furthermore, the inhibition of autophagy by 3MA (3-methyladenine) or shRNA against Atg7 (shAtg7) has been shown to lower myosin heavy chain expression and impair myoblast fusion and differentiation, suggesting that the autophagy is required during myoblast differentiation, and it has been shown to protect them from stress-induced apoptosis [72]. Furthermore, myogenesis involves an increased energetic demand of contractile myotubes and shifts from a glycolytic state to oxidative phosphorylation. This process requires dramatic remodeling of the mitochondrial network involving both mitochondrial clearance and biogenesis that is achieved by autophagy. It was reported that the autophagy inhibitors disrupt myogenic differentiation, suggesting the essential role of autophagy and mitophagy in the process [73]. In view of this, we investigated the effect of Ashwagandha extracts and the purified withanolides on autophagy by examining the marker LC3B-II. Western blotting analysis of the control and treated cells revealed an increase in LC3B-II (Figure 7B). The result was also confirmed by immunostaining by a specific anti-LC3B-II antibody (Figure 8B). In addition, the upregulation of Beclin1, ATG5, and ATG16L1, and the downregulation of p62 revealed an activation of autophagy (Supplementary Figure S2). These data suggested that Ashwagandha extracts/bioactive compounds could promote muscle differentiation by regulating hypoxia and autophagy.

**Figure 9:**
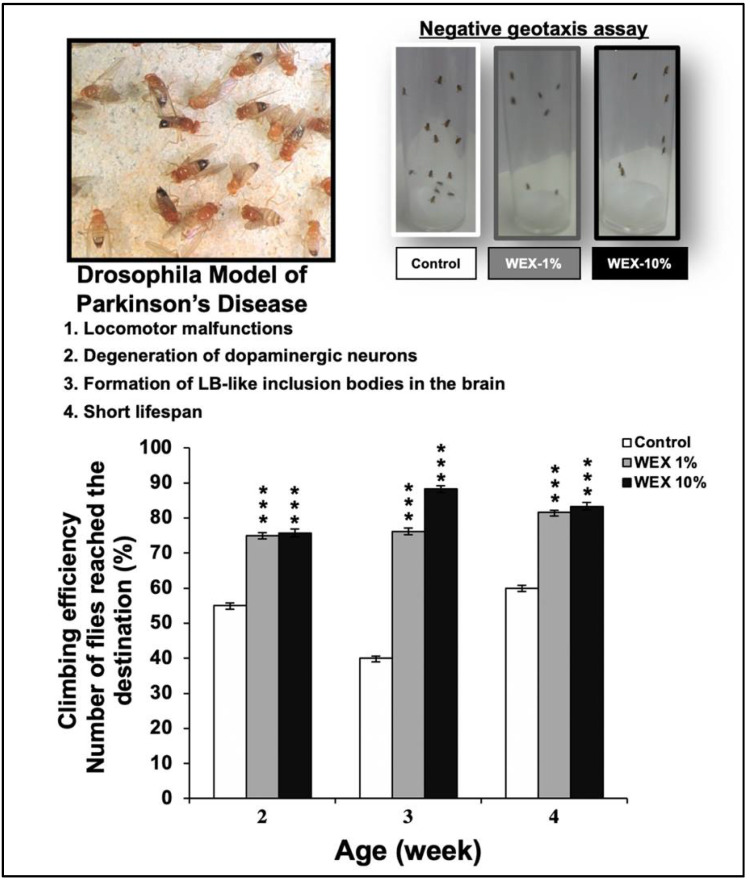
Effect of Ashwagandha water extracts (WEX) and purified withanolides on the locomotor ability of Drosophila. The negative geotaxis assay showed clear improvement in the flight activity of flies fed with Ashwagandha leaf extract. Whereas control flies showed slow upward movement and hence seen in the image, the extract-fed flies showed rapid upward movement. Quantitation of the results is shown below (mean ± SD, *n* = 3), *** *p* < 0.001 (Student’s *t*-test to control).

We next examined the potential activity of Ashwagandha leaf extract, rich in Wi-A and Wi-N, using a Parkinson’s disease model of Drosophila. These flies possessed a neuromuscular disorder and impaired flight activity. Of note, growing flies fed with the water-extract-supplemented medium showed clear improvement in their climbing and flight activity (Figure 9), suggesting the potential of Ashwagandha withanolides for the management of muscle repair and activity.

## 4. Discussion

Ashwagandha is a prominent herb, generally used for overall health promotion including stress and anxiety management, youthful vitality, activation of the immune and nervous systems, and muscle strength and endurance. Some pre-clinical trials and clinical studies also supported the therapeutic use of Ashwagandha for brain-related disorders such as anxiety, cognitive and neurological disorders, and Parkinson’s disease [2,47,49]. Wi-A, Wid-A, and Wid-N are considered as major bioactive compounds obtained from the root, stem, and leaves of Ashwagandha extracts. Wi-A isolated from roots has been shown to possess a variety of health benefits such as anti-inflammatory and anti-oxidative activities, an inhibition of OVA-induced lung injury and fibrosis, and a reduction in the infarct area and intimal hyperplasia [3,11,12,13,14,15,16]. Wi-N has been well documented in in vitro and in vivo models for its anti-stress and anti-aging activities [32,33,34,35,36,37,38]. It has also been reported that Wi-N possesses multifunctional neuroprotective effects in alleviating cognitive dysfunction by the inhibition of acetylcholinesterase (AChE), the modification of Aβ processing, and protection against oxidative stress and anti-inflammatory effects [2,3,4,16,36,37]. The anti-stress effect of Ashwagandha extracts has also been evident by studies on the biological model of animals [39,40]. The dose-related reversal of the stress effects evident by the augmentation of SOD and LPO activities and enhanced activities of CAT and GPX supported the clinical use of Ashwagandha as an antistress adaptogen [74]. Sarcopenia is a type of the loss of skeletal muscle mass, quality, and strength that occurs with aging. The herbal combination of *Boswellia serrata*, *Cissus quadrangularis*, and *Withania somnifera* on Sarcopenia has shown a significant improvement in muscle mass, grip strength, motor coordination, gait, locomotor activity, and endurance, suggesting the potential of the herbal combination to treat pathophysiological changes associated with Sarcopenia [43]. Treatment with *Withania somnifera* has shown a significant increase in lifespan, has rescued climbing impairment of ALS-Drosophila, and has exhibited neuroprotective effects on the Parkinson’s disease model of Drosophila [45,46]. Several studies have reported that Ashwagandha may improve body composition and increase strength [47,50,75]. In another study, it was reported that the people who consumed Ashwagandha regularly acquired significantly higher muscle strength and size [50]. The studies suggested the potential of Ashwagandha for increasing muscle mass and strength.

Based on the above reports, we investigated the differentiation potential and stress tolerance in response to treatment with Ashwagandha extracts, Wi-A, and Wi-N in C2C12 myoblasts. We selected a C2C12 clone (C3) with weak and uniform differentiation characteristics for the experiments. We found that a low withanolides content (Wi-A+Wi-N; 0.05 to 0.1 μM) and a high ratio of Wi-N:Wi-A (3 to 5) could result in strong differentiation of the C3 clone and recover metal-induced aggregation of the GFP protein. However, the extracts containing a relatively high level of Wi-A have a better effect on the recovery of heat-induced luciferase folding. This result may be due to the enhancement of the heat shock response triggered by Wi-A [76]. Wi-A has been shown to induce the accumulation of heat-shock proteins by inhibition of proteasome-mediated degradation, resulting in thermotolerance [20,77,78].

Skeletal muscle differentiation is a complex process that requires the activation of satellite cells that are normally resident in hypoxic areas of the tissue to maintain them in an undifferentiated state [79,80]. Generally, HIF-1α activation has been described as beneficial for the cell during hypoxic stress. Hypoxia plays a fundamental role in activating myogenesis [81]. The activation of HIF-1α promotes myogenesis through the noncanonical Wnt/β-catenin pathway [81]. The induction of myoblast differentiation requires the activation of several signaling kinases that are associated with the regulation of autophagy in skeletal muscle [82,83,84]. An increase in catabolic processes is required for the execution of the differentiation process and the formation of mature myotubes [85,86,87]. Autophagy may be rapidly induced upon myoblast differentiation to facilitate the elimination of pre-existing structures and proteins in order to promote differentiation and remodeling [72]. Consistently, our data showed that the level of expression of HIF-1α, LC3B-II, Beclin1, ATG5, and ATG16L1 that initiates hypoxia and autophagy is elevated in response to Ashwagandha extract Wi-A- and Wi-N-treated myoblasts during myoblast differentiation. On the other hand, p62 showed a decrease. These data were consistent with the previous reports showing an increase in HIF-1α [81] and LC3B-II [88] during myoblast differentiation. Taken together, the present results demonstrate that the treatment with Ashwagandha withanolides could promote muscle cell differentiation.

## 5. Conclusions

Ashwagandha-derived withanolides and extracts possess multimodal anti-stress activities. Withanone and withanone-rich extracts promote muscle differentiation suggesting their use in muscle repair and sports medicine.

## Data Availability

All datasets used and/or analyzed during the current study are available in the manuscript and Supplementary Information Files.

## Supplementary Materials

The following are available online at https://www.mdpi.com/article/10.3390/biom11101454/s1, Figure S1: Isolation of C2C12 clones with weak and uniform differentiation characteristics, Figure S2: Western blotting analysis for Beclin1, ATG5, ATG16L1 and p62 proteins (biomarkers for autophagy activation) after incubation of C2C12-C3 clone with Ashwagandha withanolides, Table S1: Determination of nontoxic concentrations of Ashwagandha extracts and purified withanolides on C2C12 cells.
