# Supplementary material for: Effect of Ashwagandha Withanolides on Muscle Cell Differentiation
Source: Biomolecules. 2021 Oct 4;11(10):1454. doi: 10.3390/biom11101454 (PMC8533065; doi:10.3390/biom11101454)

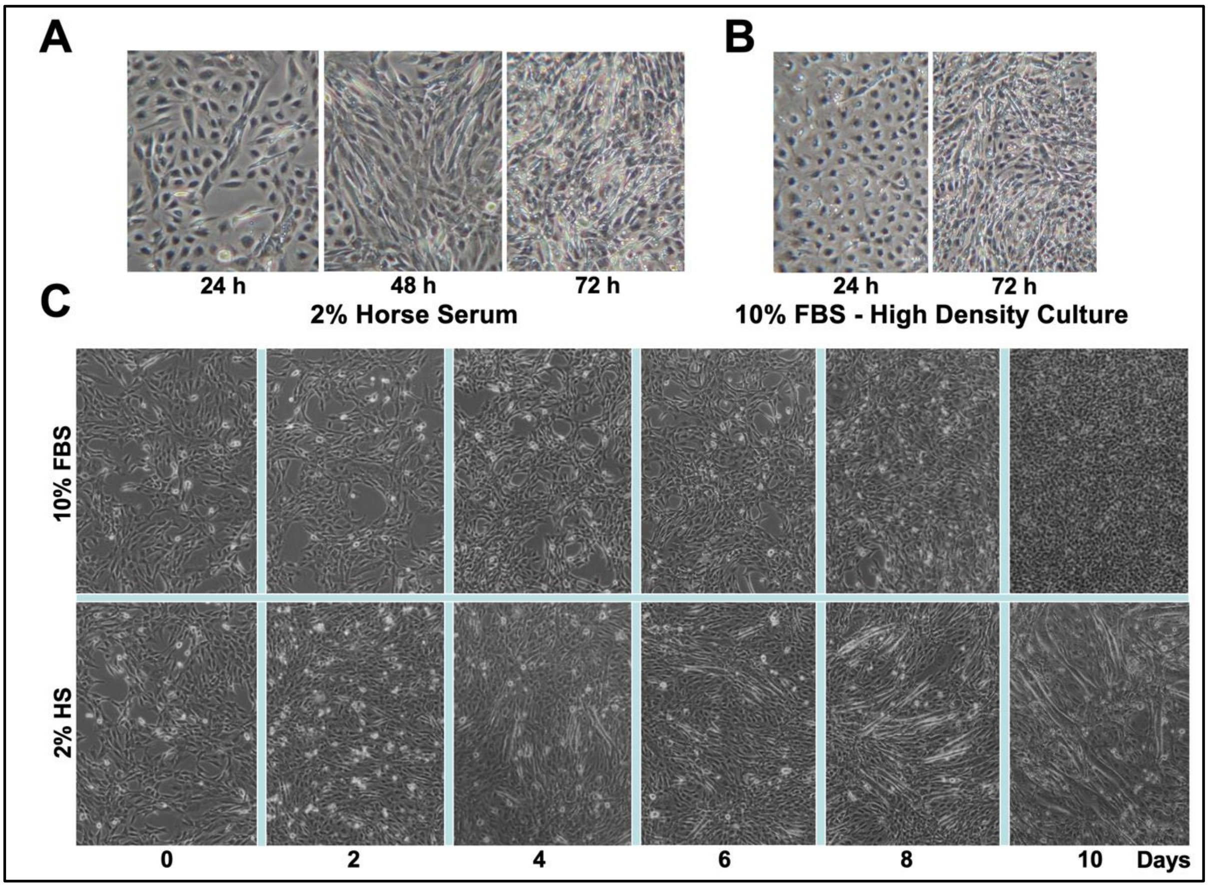

Figure S1. Isolation of C2C12 clones with weak and uniform differentiation characteristics. C2C12 myoblasts were differentiated by culturing them in medium-supplemented with 2% horse serum (HS) (A) and high density culture in medium-supplemented with 10% FBS (B). They showed heterogenous differentiation. (C) C3 clone of C2C12 myoblasts showed differentiation into myotubes in 2% HS supplemented medium, but not in 10%FBS high density culture.

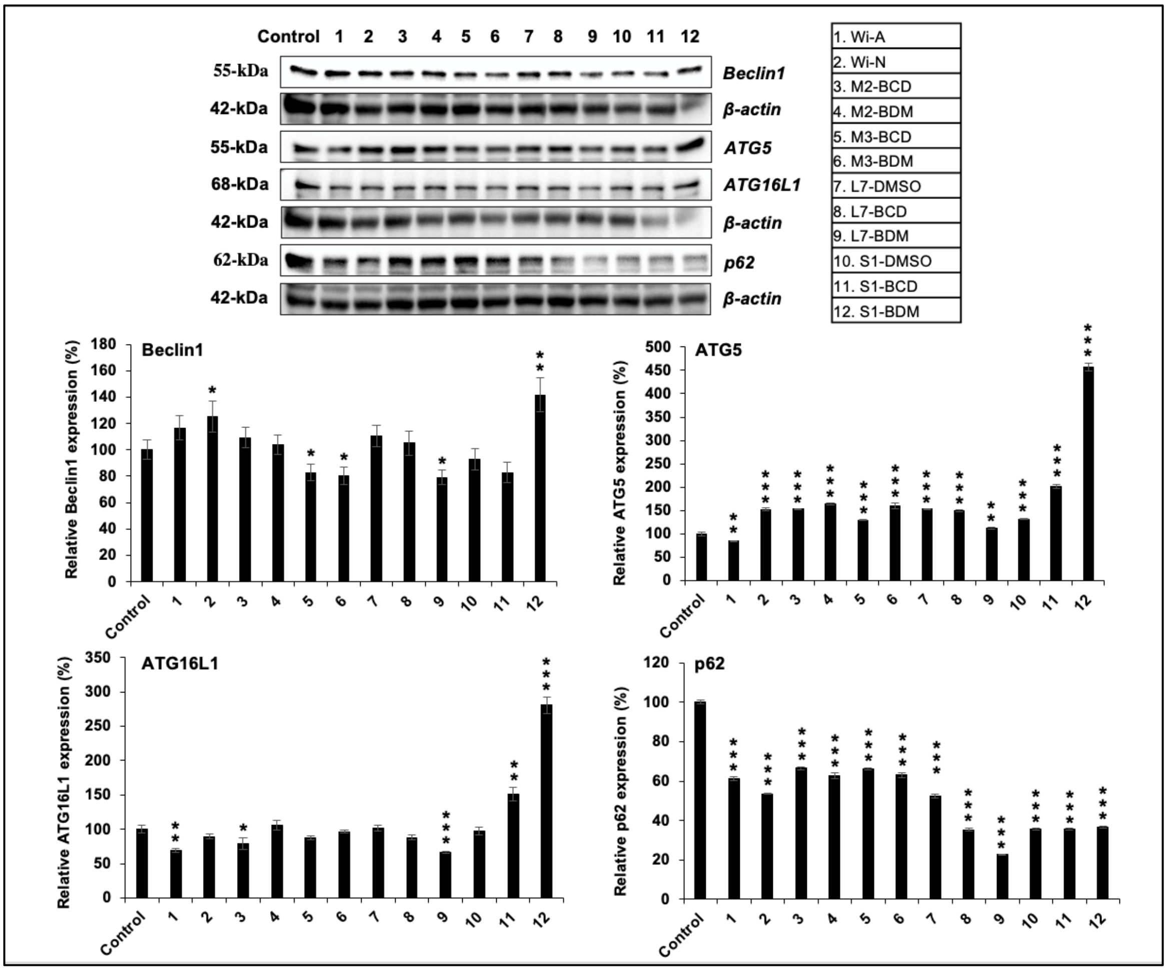

Figure S2. Western blotting analyses for Beclin1, ATG5, ATG16L1 and p62 proteins (biomarkers for autophagy activation) after incubation of C2C12-C3 clone with Ashwagandha withanolides. Quantitation of the results is shown below (mean ± SD, n = 3), **p*<0.05, ***p*<0.01, ****p*<0.001 (Student's t-test to control).

Table S1. Determination of nontoxic concentrations of Ashwagandha extracts and purified withanolides for C2C12 cells.

	Wi-A	Wi-N	M2-BCD	M2-BDM	M3-BCD	M3-BDM	L7-DMSO	L7-BCD	L7-BDM	S1-DMSO	S1-BCD	S1-BDM
IC50	1 µM	40 µM	1%	1%	1%	1%	0.3%	0.3%	0.3%	2%	2%	2%
Nontoxic doses	0.1 µM	5 µM	0.1%	0.1%	0.1%	0.1%	0.05%	0.05%	0.05%	0.1%	0.1%	0.1%

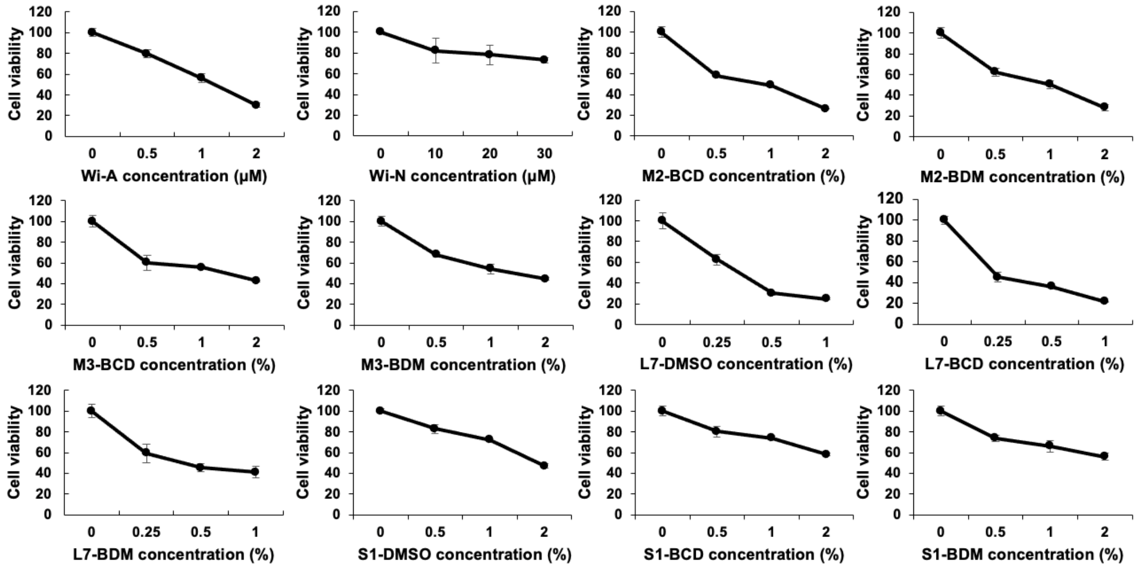

Supplement: Supplementary file 1 [file biomolecules-11-01454-s001.zip › biomolecules-1344729-supplementary.pdf]
